# GRANDPARENTING AND DEPRESSIVE SYMPTOMS IN URBAN CHINA: LIVING ARRANGEMENTS AND WORK STATUS AS MODERATING EFFECTS

**DOI:** 10.1093/geroni/igac059.1890

**Published:** 2022-12-20

**Authors:** Luyan Ma, Zhaowen Cheng

**Affiliations:** Peking University, Beijing, Beijing, China (People's Republic); China Development Research Foundation, Beijing, Beijing, China (People's Republic)

## Abstract

Scholars worldwide are divided as to whether grandparenting can benefit to older adults’ well-being. Evidence from rural China supports that custodial grandparenting worsens grandparents’ psychological well-being. However, given the great urban-rural gap, the situation in urban China might be different. To clarify this, we use CHARLS 2011-2013 (900 respondents) and examine the association between length and intensity of grandchild care and depressive symptoms among older adults living in urban China. We also explore the moderating effect of grandparents’ living arrangements and work status. Multiple linear regression with interaction analysis is applied in our analysis. We find that moderate levels of grandchild care, without distinguishing between living arrangements and work status, are associated with reduced depressive symptoms (β = -1.146，p < .05). Grandparents are likely to have more depressive symptoms when working full-time and providing care at the same time. Comparing with not living with grandchildren, living with grandchildren is beneficial to grandparents’ mental health. Findings suggest that psychological counseling services and economic support policies should be launched to relieve the work pressure of grandparents in financial difficulties.

